# Identification of a miRNA-based non-invasive predictive biomarker of response to target therapy in BRAF-mutant melanoma

**DOI:** 10.7150/thno.77761

**Published:** 2022-10-24

**Authors:** Ciro Francesco Ruggiero, Luigi Fattore, Irene Terrenato, Francesca Sperati, Valentina Salvati, Gabriele Madonna, Mariaelena Capone, Fabio Valenti, Simona Di Martino, Chiara Mandoj, Domenico Liguoro, Vittorio Castaldo, Giordana Cafaro, Ester Simeone, Vito Vanella, Michelangelo Russillo, Laura Conti, Giovanni Cuda, Diana Giannarelli, Paolo Antonio Ascierto, Rita Mancini, Gennaro Ciliberto

**Affiliations:** 1 Department of Melanoma, Oncologic Immunotherapy and Innovative Therapies, Istituto Nazionale Tumori-IRCCS-Fondazione G. Pascale, Napoli, Italy.; 2 SAFU Laboratory, Department of Research, Advanced Diagnostics and Technological Innovation, Translational Research Area, IRCCS Regina Elena National Cancer Institute, Rome, Italy.; 3 UOSD Clinical Trial Center e Biostatistica e Bioinformatica, IRCCS Regina Elena National Cancer Institute, Rome, Italy.; 4 San Gallicano Dermatological Institute IRCCS UOSD Clinical Trial Center e Biostatistica e Bioinformatica, Scientific Direction Via Elio Chianesi 53, 00144, Rome, Italy; 5 Department. Preclinical Models and New Therapeutic Agents Unit, IRCCS Regina Elena National Cancer Institute, Rome, Italy.; 6 Oncogenomic and Epigenetic Unit, Department of Diagnostic Research and Technological Innovation, IRCCS Regina Elena National Cancer Institute, Rome, Italy.; 7 Biological Tissue and Liquid Bank, Scientific Direction, IRCCS Regina Elena National Cancer Institute, Rome, Italy.; 8 Clinical Pathology, IRCCS Regina Elena National Cancer Institute, Rome, Italy.; 9 Department of Molecular and Clinical Medicine, University of Rome “Sapienza”, 00161 Rome, Italy.; 10 Department of Clinical and Molecular Medicine, Sapienza University of Rome, Rome, Italy.; 11 Division of Medical Oncology1, IRCCS Regina Elena National Cancer Institute, Rome, Italy.; 12 Department of Experimental and Clinical Medicine, University “Magna Graecia” of Catanzaro, Catanzaro, Italy.; 13 Scientific Direction, IRCCS Regina Elena National Cancer Institute, Rome, Italy.

**Keywords:** microRNA, liquid biopsy, biomarkers, metastatic melanoma, target therapy.

## Abstract

**Rationale:** Metastatic melanoma is the most aggressive and dangerous form of skin cancer. The introduction of immunotherapy with Immune checkpoint Inhibitors (ICI) and of targeted therapy with BRAF and MEK inhibitors for BRAF mutated melanoma, has greatly improved the clinical outcome of these patients. Nevertheless, response to therapy remains highly variable and the development of drug resistance continues to be a daunting challenge. Within this context there is a need to develop diagnostic tools capable of predicting response or resistance to therapy in order to select the best therapeutic approach. Over the years, accumulating evidence brought to light the role of microRNAs (miRNAs) as disease biomarkers.

**Methods:** In particular, the detection of miRNAs in whole blood or specific blood components such as serum or plasma, allows these molecules to be good candidates for diagnosis, prognosis and for monitoring response to anticancer therapy. In this paper, we evaluated circulating basal levels of 6 previously identified miRNAs in serum samples of 70 BRAF-mutant melanoma patients before starting targeted therapy.

**Results:** Results show that the circulating levels of the oncosuppressor miR-579-3p and of the oncomiR miR-4488 are able to predict progression free survival (PFS) but not overall survival (OS). Most importantly, we observed that the best predictor of disease outcome is represented by the ratio of circulating miR-4488 *vs.* miR-579-3p (miRatio). Finally, the combination of the Lactate dehydrogenase (LDH) blood levels with the two circulating miRNAs alone or together did not produce any improvement in predicting PFS indicating that miR-579-3p and miR-4488 are independent predictors of PFS as compared to LDH.

**Conclusions:** All together these data underscored the relevance of circulating miRNAs as suitable tools to predict therapy response in melanoma and maybe further developed as companion diagnostics in the clinic.

## Introduction

The treatment of metastatic melanoma has seen considerable progress in recent years thanks to the advent of targeted therapy and of immunotherapy with immuno-checkpoint inhibitors (ICI) 1, 2. For targeted therapy the gold standard consists in the combination therapy with BRAF and MEK inhibitors (MAPKi) directed at blocking the BRAF/MEK/MAPK signaling pathway in BRAFV600 mutated melanomas. Although this approach provides significant benefit in terms of objective responses, time to progression and overall survival, the effects are often omitted by innate or acquired drug resistance which results in tumor relapses with a median time-to-recurrence of approximately 12-15 months 2, 3. Furthermore, relapsing tumors are highly aggressive and scarcely treatable 4, 5. Of note, although immunotherapy is often utilized as a salvage option after disease progression to MAPKi in BRAF-mutated melanoma patients, important studies suggest that acquired resistance to MAPK-targeted therapy 6 is a negative factor for subsequent response to immunotherapy 7-10. Based on these important assumptions it is necessary to develop new diagnostic tools capable of predicting which therapy might offer the best benefit in terms of response in advanced melanoma patients.

Tissue biopsy is the current "golden standard" for cancer diagnosis and evaluation. However, the surgical removal of tumor tissue is considered rather an invasive procedure and is associated with several limitations 11-13. The most relevant of these, consists in its incompatibility with collecting longitudinal samples over time. In line with this, the advent of liquid biopsies represents a huge opportunity in overcoming these limitations. This approach allows to monitor longitudinally and in a non-invasive manner patient responses to specific treatments and decide whether to continue the therapy or promptly change it. 13, 14. Several lines of evidence have highlighted the potential use of circulating molecular biomarkers for melanoma diagnosis and prognosis. Among them, circulating tumor cells (CTCs), cell-free circulating tumor DNA (ctDNA), lactate dehydrogenase (LDH), S100 calcium-binding protein B (S100B) and the cell-free circulating RNA (cfRNA), were found to be suitable monitoring factors in advanced melanoma patients 11, 15-20. In addition, also FDA approved diagnostic panels to monitor minimal residual disease in Stage II and III colorectal cancer to guide therapeutic decisions 11. Among the cfRNA, growing relevance has been attributed to a class of small non-coding RNA called microRNAs (miRNAs). Inside the cells, these small molecules are essential for regulating post-transcriptional gene expression and where their altered balance is associated with several pathological conditions including cancer 15, 21-25. Furthermore, the alteration of miRNA expression has been correlated with the establishment of drug resistance 26-28. MiRNAs, can be released into and circulate via biological fluids in association with proteins such as AGO2 or alternatively can be encapsulated with high stability into extracellular vesicles (EVs) such as exosomes 29-31. Of note, although miRNAs are present in low concentrations in the bloodstream, their expression can be easily detected through using the standard real-time quantitative reverse transcription polymerase chain reaction (qRT-PCR). For these reasons, circulating miRNAs are increasingly emerging as ideal non-invasive biomarkers to diagnose the development of many cancers including melanoma. 32-37.

It is important to address two issues regarding circulating miRNAs i.e. normalization and quantification. The first problem derives from evidence showing that there are no circulating miRNAs that can act as reference genes. The canonical small nuclear RNAs used as reference genes in cellular/tissue samples, such as RNU48 and RNU6, are not suitable for normalizing extracellular miRNAs due to RNase-mediated degradation 24. The second issue regarding total RNA extracted from serum/plasma samples is usually below the threshold of standard quantification methods such as UV spectrophotometry or fluorescence-based spectrophotometry. Based on these aspects, a fixed volume is often chosen as the input for the reverse transcription reaction (RT), and therefore, subsequently statistical algorithms (Global Mean, NormFinder etc) are used to analyze the results obtained from qRT-PCR 15. The Global Mean method attributes equal weight to each individual miRNA during normalization and requires the calculation of the Ct mean among the candidates evaluated. This value is used as a reference to normalize expression levels of each miRNA candidate as in a canonical qRT-PCR 15. Conversely, the NormFinder tool is used to establish the miRNA that suffers less fluctuations among those analyzed. Once identified, the miRNA is used as a reference to normalize and calculate the expression of the others 15. Alternatively, another approach used to normalize miRNA levels is to calculate the expression ratio of different miRNAs (miRatio) as already reported in several publications 38-40. For this kind of analysis, it is important to consider those miRNAs that show an opposite trend of expression. Thanks to this approach, in several diseases such as lung cancer and diffuse large B-cell lymphoma (DLBCL) it was possible to identify the diagnostic and prognostic value of miRNA as biomarkers 38-39.

In recent years, our laboratory has intensively studied the role of miRNAs in melanoma progression and resistance to BRAF/MEK inhibitors 27, 41-43. Initially, we identified the involvement of the oncosuppressor miR-579-3p. This miRNA was found to be down-regulated in BRAF-mutant melanoma cells and even more when they developed resistance to MAPKi. Furthermore, its down-modulation was also confirmed in matched tumor samples from patients before and after developing resistance to targeted therapies 41. This study was followed by a comprehensive analysis of the changes involving the entire miRNome during the development of resistance to MAPKi. This led to the identification of more than 20 disregulated miRNAs and to a thorough characterization of five of them 42. In particular, three miRNAs (miR-9-5p, miR-4443 and miR-4488) resulted strongly up-regulated whereas the other two miRNAs were found downregulated (miR-199b-5p and miR-204-5p) 42-44 during the development of drug resistance. These miRNAs were found significantly modulated also in solid and liquid biopsies of melanoma patients after disease recurrence 42. Of note, we confirmed that miR-199b-5p expression levels were down-regulated in the plasma of 25 melanoma patients post-MAPKi treatment as compared to the plasma from untreated patients. An opposite trend was obtained for miR-4488 42. These data prompted us to further assess the potential of these miRNAs as predictors of response to therapy. To this aim, we conducted the present study on a retrospective cohort of 70 patients treated with MAPKi therapy as first line therapy.

## Results

### Circulating levels of miR-579-3p and miR-4488 distinguish BRAF melanoma patients who could benefit from target therapy

In this retrospective study, we analyzed serum samples from 70 BRAF-mutant melanoma patients coming from two cancer centers namely the National Cancer Institute IRCCS "G. Pascale Foundation" in Naples and the IRCCS Regina Elena National Cancer Institute in Rome (IRE). The general characteristics of the 70 BRAF-mutated melanoma patients are summarized in Table 1. The complete databases containing the information for all 70 recruited melanoma patients, are present in the table S4 and S5 for the National Cancer Institute IRCCS "G. Pascale Foundation" and in the table S6 for the IRCCS Regina Elena National Cancer Institute. All these patients with advanced melanoma were treated with MAPKi (either BRAFI monotherapy or combo therapy with a BRAF and a MEK inhibitor) as first line therapy. Circulating microRNAs were extracted from basal serum samples (before starting therapy) according to procedures described in the material and methods section. MiRNA levels were analyzed by qRT-PCR. Six miRNAs previously identified by our group were analyzed, three oncomiRs namely miR-9-5p, miR-4443 and miR-4488 and three oncosuppressor miRNAs, i.e. miR-199b-5p, miR-204-5p, miR-579-3p) 41, 42.

**Figure 1:**
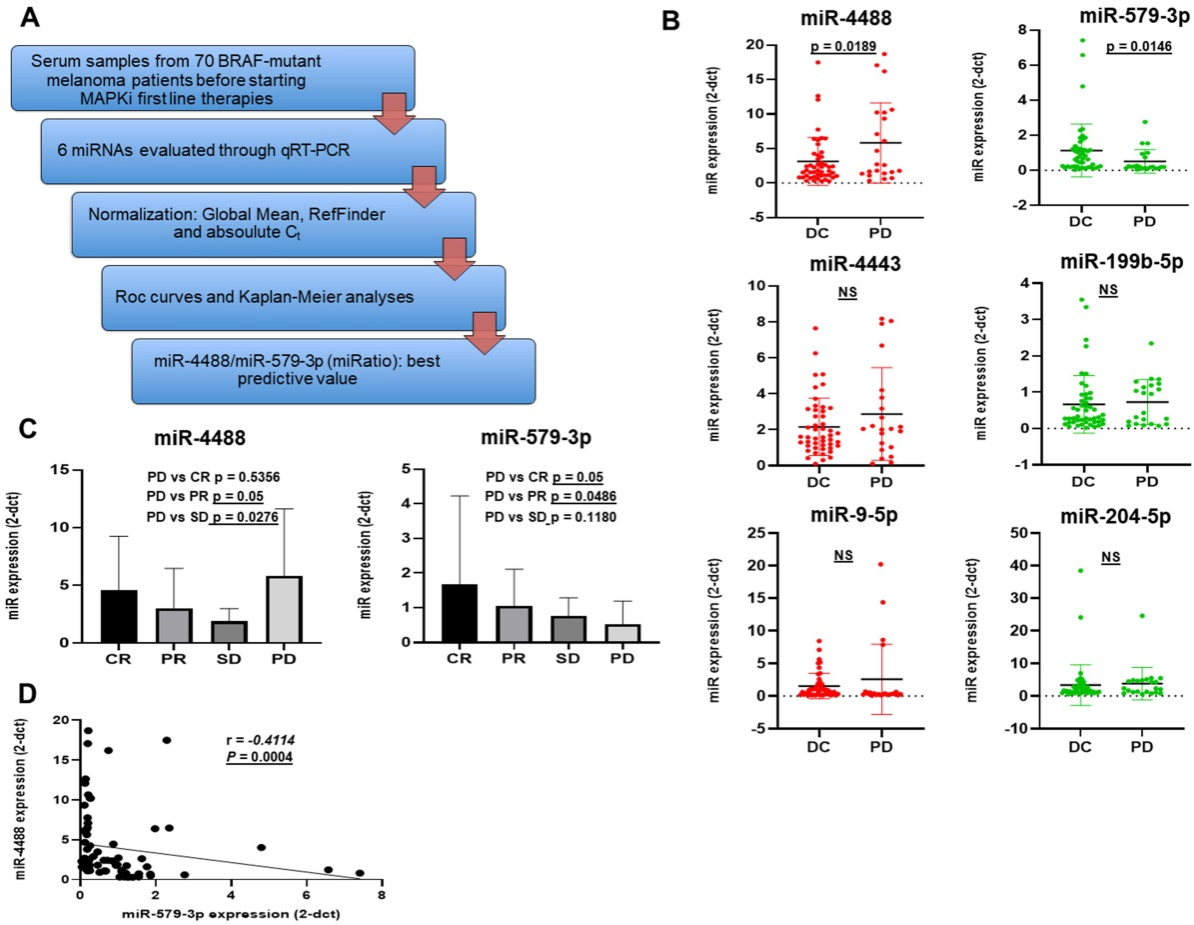
Part A summarizes the workflow of the retrospective study conducted on serum sample from 70 BRAF-mutant melanoma patients. Box plot graphs based on miRNA expression show that miR-4488 (oncomiRNA) and miR-579-3p (oncosuppressor miRNA) were the only significantly deregulated between melanoma patients had achieved a disease control (DC: stable disease, partial/complete response) as best tumor response, and those who underwent disease progression (PD) before starting therapy (B). Moreover we observed that the expression of miR-579-3p progressively decreased among patients splitted according the four RECIST criteria i.e. CR, PR, SD and PD (C, right panel). In contrast the highest expression levels of miR-4488 were found in basal serum samples of patients who undergo rapid disease progression to MAPKi treatment (PD) (C, left panel). Finally, a significant negative Spearman correlation was obtained between the circulating levels of miR-4488 vs miR-579-3p in basal serum samples (D).

Given that no reference miRNAs are available in the serum, data of circulating miRNAs were normalized using two different methods: the Global Mean Normalization (GMN) 42 and the RefFinder, a web-based comprehensive tool developed for evaluating and screening reference genes from extensive experimental datasets. In addition, we also decided to examine cycle threshold values (Ct values) for our analysis as shown subsequently (Figure 1A). First of all, we decided to artificially divide patients according to the Response Evaluation Criteria in Solid Tumors (RECIST 1.1) 45 in two groups. Patients belonging to the first group (DC for Disease Control) experienced as best objective response either complete response (CR), partial response (PR) or stable disease (SD whereas the second group is composed by patients who developed a progressive disease (PD) within 6 months from the start of therapy. 46, 47. The expression levels of each miRNA were expressed in terms of 2^-dCt values obtained through GMN 42 (Figure 1B). Among the 6 miRNAs analyzed, the results revealed that only miR-4488 and miR-579-3p showed a significantly different expression (*p* < 0.05) between the two groups. In particular, melanoma patients undergoing fast PD were characterized by higher circulating levels of miR-4488 as compared to patients experiencing DC. miR-579-3p levels showed the opposite trend. The other miRNAs evaluated were not able to significantly distinguish patients with PD from. DC patients. Based on these results, we decided to focus further studies on these two miRNAs. We further subdivided the 70 melanoma patients into the following four groups according to the RECIST criteria: i.e. CR = complete response; PR = partial response; SD = stable disease; PD = progressive disease and evaluated the distribution of miR-4488 and miR-579-3p (Figure 1C). In detail each group is composed from 24 CR, 12 CR, 12 SD and 22 PD. Interestingly, we observed that miR-579-3p expression progressively decreased moving from CR to PD patients (Figure 1C right panel). A contrasting trend was observed for miR-4488, where the highest expression levels were found in basal serum samples of patients who underwent rapid disease progression to MAPKi treatment (Figure 1C left panel). Finally, we observed a significant negative Spearman correlation between the circulating levels of miR-4488 vs. miR-579-3p in basal samples (Figure 1D).

Hence, we can conclude from these data that the circulating levels of the oncosuppressor miR-579-3p and of the oncomiR miR-4488 are able to identify BRAF-mutated melanoma patients who benefit or not from target therapy with BRAF and MEK inhibitors.

### The relative ratio of circulating mir-4488 vs. miR-579-3p predicts response to targeted therapy in BRAF-mutant melanoma patients

**Figure 2:**
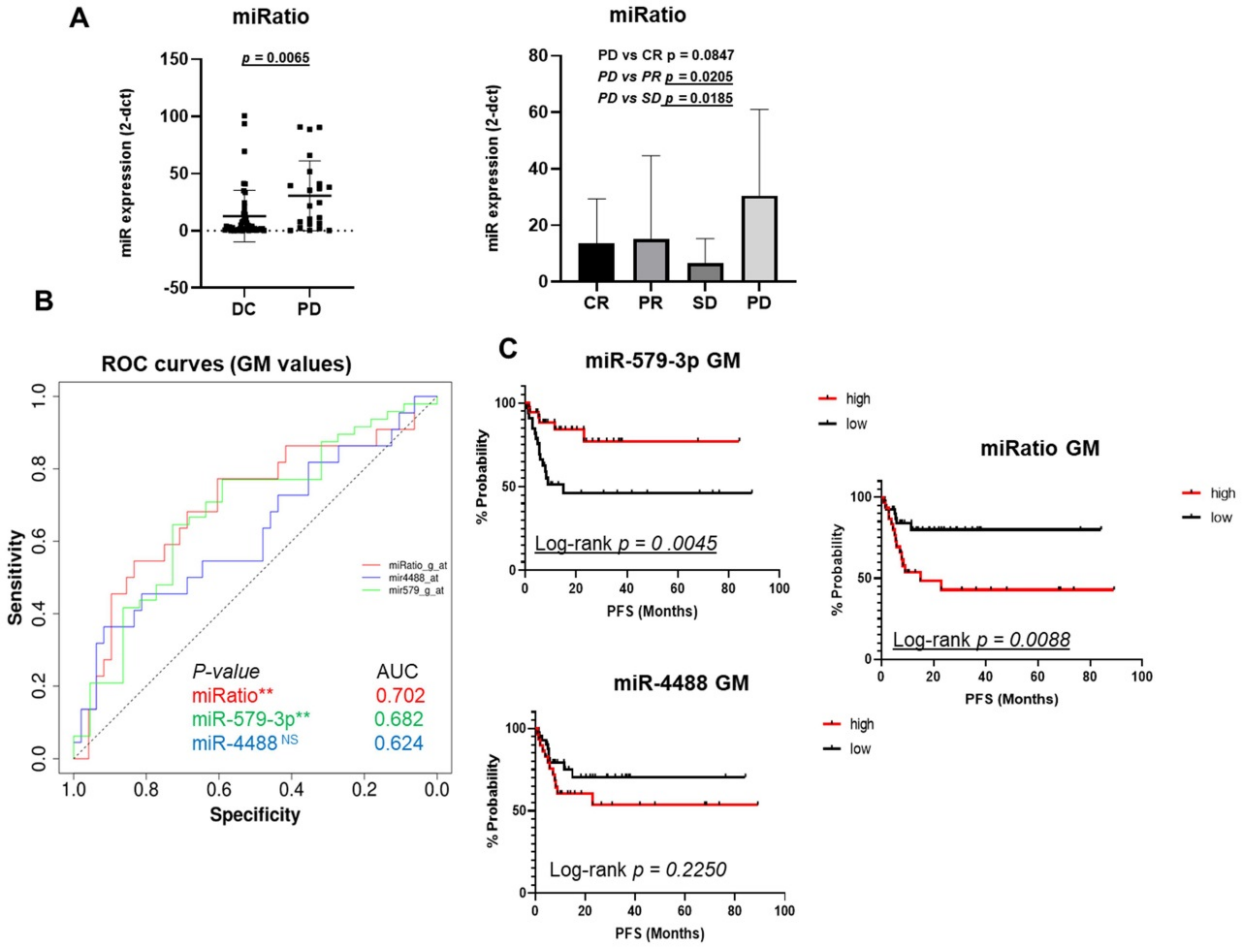
The miRatio significantly (p=0.0065) distinguished BRAF-mutated melanoma patients characterized by DC vs PD responses (A, left panel). These findings were significantly confirmed also splitting the patients into the four RECIST categories (CR, PR, SD and PD) (A, right panel). The ROC curve shows that miRatio owns the best predictive value as demonstrated by the highest AUC value, i.e. 0.702, as compared to the individual miRNAs (B, red line). The Kaplan-Meier curve clearly show that high expression levels of miR-579-3p before starting therapy are a predictive factor of better PFS in metastatic melanoma patients (2C, red line upper panel) as compared to patients with lower levels of this miRNA (2C, black line upper panel). An opposite result was obtained with miR-4488 (2C, bottom panel). Finally, Kaplan-Meier curves plotted using miRatio values also confirmed a significant PFS prediction (C, right panel).

One useful approach used to evaluate the expression levels of miRNA in biological fluids is determining their expression ratio especially when anti-correlated candidates are being examined 38, 39. To this purpose, we calculated the relative ratio of the circulating levels of miR-4488 vs. miR-579-3p (from here simply miRatio) based on GMN values. Results clearly showed that miRatio significantly distinguished BRAF-mutated melanoma patients characterized by DC vs. PD (P < 0.05). In particular, we observed higher levels of this parameter in basal samples deriving from patients who developed faster PD. These data suggest that patients characterized by circulating miR-4488 levels which dominate over miR-579-5p levels had the worst therapeutic response to MAPKi (Figure 2A left panel). These findings were confirmed also when splitting the patients into the four categories according to RECIST criteria, i.e. CR, PR, SD and PD (Figure 2A right panel).

The predictive potential of the combination of miR-4488 upregulation and miR-579-3p downregulation was further assessed by constructing receiver operating characteristic (ROC) curves. The parameters of sensitivity, specificity and accuracy were evaluated together in order to assess the Area Under Curve (AUC) and to evaluate the performance of each classifier in a single measure. In particular, miR-579-3p yielded an AUC value of 0.682 (Figure 2B, green line), whereas miR-4488 of 0.624 (Figure 2B, blue line). Pleasingly, we observed that the miRatio between the two miRNAs owned the best predictive value as demonstrated by the highest AUC value, i.e. 0.702, as compared to the individual miRNAs (Figure 2B, red line)* (p-value<0.5)*.

The cut-off calculated through the ROC curves was used to categorized miRNA values in order to generate the Kaplan-Meier curves to estimate whether basal miRNA expression levels may be predictive of Progression-free survival (PFS in months). Results from the Kaplan-Meier analysis clearly showed that high expression levels of miR-579-3p before starting therapy are a predictive factor of better PFS in metastatic melanoma patients (red line Figure 2C upper panel) as compared to patients with lower levels of this miRNA (black line Figure 2C upper panel). A contrasting result was obtained for miR-4488 as shown in Figure 2C, bottom panel. Most importantly, Kaplan-Meier curves plotted using miRatio values also confirmed a significant PFS prediction (Log-rank p=0.0088) (Figure 2C right panel). A summary of values used to generate the graphs are reported in table S1. We then wondered whether circulating levels of these miRNAs may predict also Overall Survival (OS) for melanoma patients (Figure S1A and Figure S1B). Results revealed that miR-579-3p, miR-4488 and miRatio were not able to predict overall survival thus suggesting their specificity as a parameter to monitor only PFS.

Finally, to further strengthen these results we also tested another normalization method, i.e. the ReFinder normalization which works by comparing various normalization methods to determine which gene may be the least fluctuating to be used as a reference. When considering the expression levels of the six miRNAs, we observed that circulating levels of miR-199b-5p were the most stable among the 70 serum samples tested, therefore becoming the reference miRNA to perform our analyses (Figures S2A and S2B). Interestingly, ROC curves confirmed also in this case and in agreement with GMN results, that the miRatio yielded the best AUC value (0,702) (Figures S2C). These results were used to categorize miRNA values and applied Kaplan-Meier method to generate curves which confirmed that the higher values of miRatio predict a worst PFS as compared to lower levels of this parameter (Figure S2D). A summary of values used to generate the graphs by the RF method is reported in table S2.

In conclusion, the ratio of miR-4488/miR-579-3p is able to predict response to targeted therapy in melanoma patients.

### Also absolute miRNA expression values are able to predict the development of drug resistance

**Figure 3:**
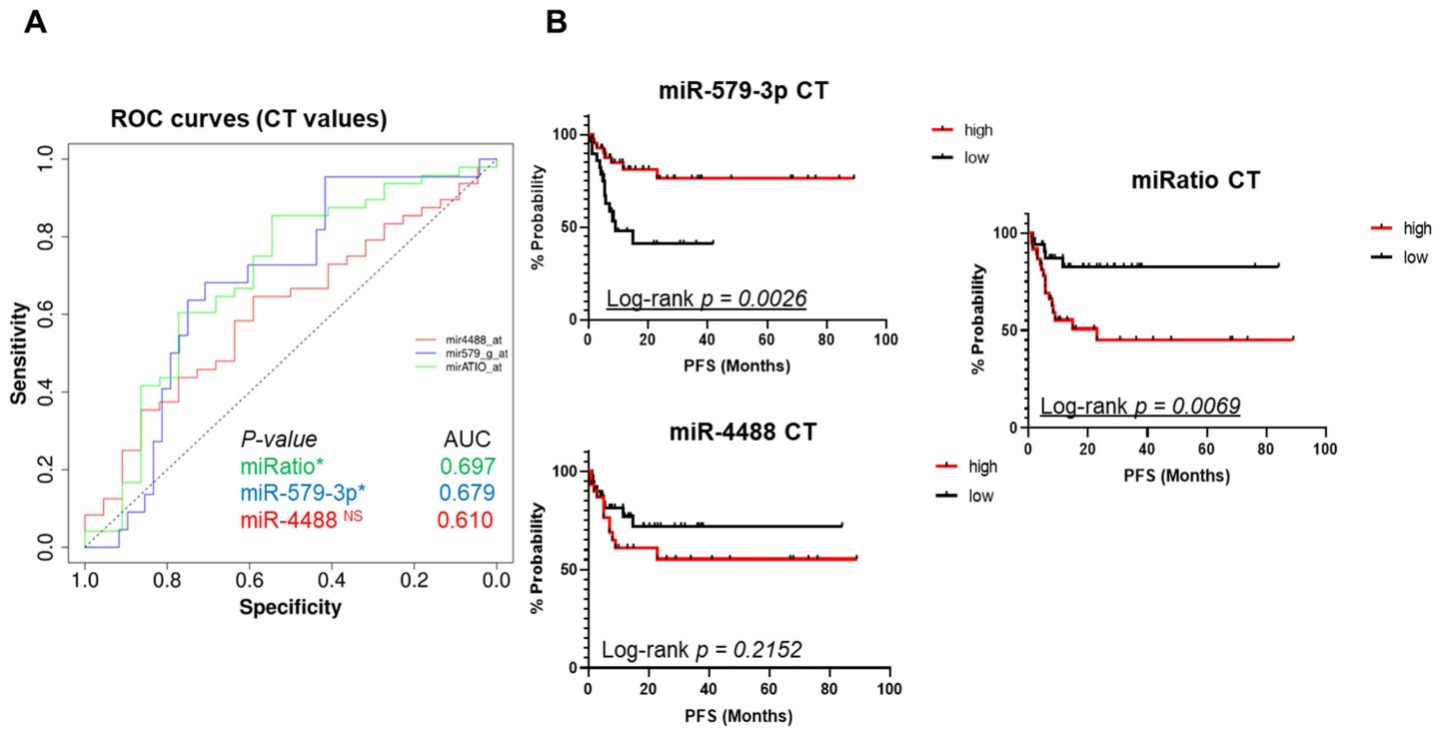
Ct absolute values were used to plot ROC curves. Also in this case the miRatio generated a ROC curve with the highest AUC value (0,697) and a better statistical significance (3A, red line). Kaplan-Meier curves plotted using ROC cut-offs confirmed that higher circulating levels of miR-579-3p predicted a better PFS (3B, upper panel). On the opposite, higher levels of miR-4488 were correlated with worst PFS in melanoma patients (3B, bottom panel). Furthermore, also in this case miRatio value obtained (by using the CT values) was in line with the previous results of GM and RF normalization methods (3B, right panel). The last figure shows bar graphs where melanoma patients were separated by CT cut-off values derived from ROC curves. The group of patients with CT cut-off values below median, i.e. the one composed of high miR-579-3p expression levels, is enriched for longer PFS as compared to the group of patients with CT cut-off values above median (3C left panel). An opposite trend was found regarding miR-4488 (3C, right panel).

Next, we decided to assess the cycle threshold values (Ct values), i.e. the absolute expression levels of miR-579-3p and miR-4488 as well as their ratio. The goal was to determine whether these parameters may also serve to predict response to targeted therapy. This is of interest because in clinical practice this would be more advantageous as compared to normalization vs. a set of given miRNAs and the results will be easier to interpret. Again, ROC analysis was applied to identify a specific cut-off in order to generate Kaplan-Meier curves to test the potential value of these miRNAs. As shown in Figure 3A, single miRNAs measurements resulted in encouraging AUC values. In addition, also in this case the miRatio (based on absolute Ct values) generated a ROC curve with the highest AUC value (0,697) and a better statistical significance *(p-value < 0,05* (Figure 3A, green line). Once again, the Kaplan-Meier curves confirmed that higher circulating levels of miR-579-3p predicted a better PFS (Figure 3B, upper panel). As expected, higher levels of miR-4488 were correlated with worst PFS in melanoma patients (Figure 3B, bottom panel). In addition, the miRNA ratio also obtained by using the CT values confirmed the previous findings (Figure 3B, right panel).

Finally, these results were also plotted as bar graphs where melanoma patients were separated by CT cut-off values derived from ROC curves in the two groups of high or low expressors of miR-579-3p or miR-4488, respectively. The resulting graphs, show that the group of patients with CT cut-off values below the median, i.e. the one composed of high miR-579-3p expressors is enriched for longer PFS as compared to the group of patients with CT cut-off values above median (Figure S3A). The reverse is true for miR-4488 (Figure S3B). Data obtained by CT values are summarized in table S3

### miR-579-3p and miR-4488 are better predictors of PFS as compared to LDH

**Figure 4:**
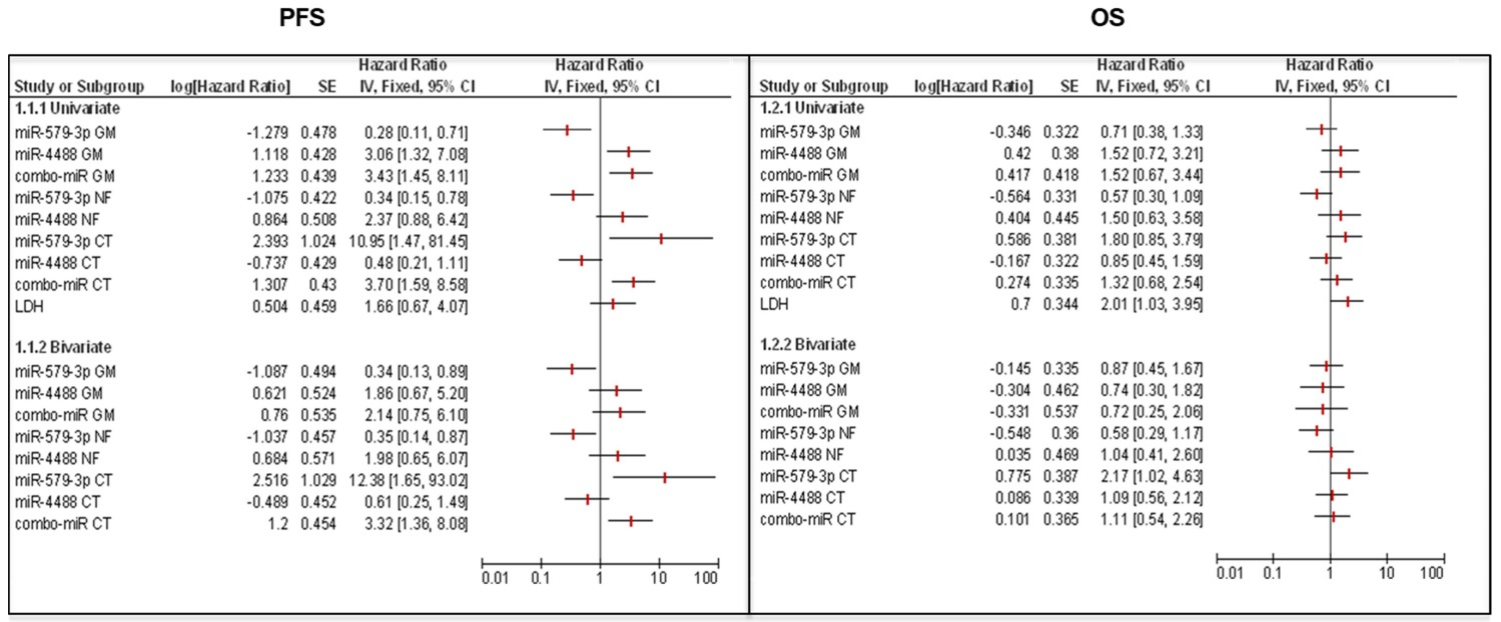
Forest Plot graphs show that the combination of LDH with circulating miRNAs (miR-579-3p and miR-4488) alone or together did not improve the Hazard Ratio (HR) for predicting PFS (left panel). Moreover, the modulation of the two miRNAs is not correlated with the overall survival (OS) both in the univariate study and in the bivariate study in combination with LDH (right panel).

In the last part of this study, we carried out a univariate and multivariate analysis to assess the predictive value of miRNA levels alone or in combination with the measure of lactate dehydrogenase (LDH) levels in blood. It is well known that the baseline LDH value represents one of the main prognostic factors associated with overall survival of patients with advanced melanoma 48, 49. According to our previous findings, the univariate analyses showed a significant effect size of miR-579-3p, miR-4488 and miRatio to predict PFS in all subgroups of studies considered (GM, RF and CT). Briefly, the Hazard Ratio value (HR) assumes values > 1 for oncogenic miR-4488 indicating that its expression is a risk factor in PFS. Conversely, for the oncosuppressor miR-579-3p, the HR resulted < 1 classifying it as a protective factor. In contrast, LDH was found to not be correlated with PFS in the univariate analysis as shown by the confidence interval. This aspect was confirmed also by the ROC and Kaplan-Meier curves (Figure S4A). Furthermore, considering the multivariate models, we observed that the combination of LDH with circulating miRNAs alone or together did not produce any improvement in the HR for predicting PFS (Figure 4 panel left). Finally, Forest Plots showed that the modulation of miR-579-3p and miR-4488 did not correlate with OS both in the univariate study and in combination with LDH (bivariate study) (Figure 4, right panel). It is important to point out that, in agreement with other published data 11, 49, 50, the modulation of LDH alone produced a significant correlation with the OS. In line with this, the ROC curve showed significant AUC value of 0,647 and the Kaplan-Meier graph displayed that high levels of LDH in basal serum samples correlate with a worst OS (Log-rank p=0.0379) (Figure S4B).

## Methods

### Experimental design

Metastatic BRAF-mutated melanoma patients (n = 36 male and n = 34 female) were enrolled in this retrospective study. All of them were treated with inhibitors of the MAPK pathway, i.e. vemurafenib or dabrafenib (as BRAF inhibitors) alone or in combination with cobimetinib or trametinib, respectively (as MEK inhibitors) between April 2013 and February 2019. Serum samples (1ml) from all patients before starting MAPK therapy were collected and preserved at the the National Cancer Institute IRCCS “G. Pascale Foundation”, Naples, Italy and the IRCCS Regina Elena National Cancer Institute, Rome, Italy, tumor biobanks.

The use of human samples was approved by Istituto Pascale's Ethical Committee with the protocol DSC/1504 on June 11, 2014 and DSC/2893 on April 11, 2015 and the IRCCS Regina Elena National Cancer Institute in Rome (IFO) Ethical Committee with the protocol 8393 of 23.07.2017. All patients signed an informed consent. Patients eligible for inclusion in this study were candidates for therapy with BRAF inhibitors and MEK inhibitors. All melanoma patients were ≥ 18 years old and were able to understand and willingly sign the informed consent form which had been submitted to their attention.

The diagnosis was confirmed as locally advanced stage melanoma histology or metastatic (stage IIIB IIIC or IV according to the American Joint Committee on Cancer - (AJCC, 7th edition) staging 51).

### Isolation and evaluation of cfMRNAs

Circulating miRNAs were extracted from the serum of 70 melanoma patients before the beginning of therapy through using the miRNeasy Mini Kit (Qiagen) according to the manufacturer's instructions. Since the concentration of total RNA was undetectable either when using the Nanodrop system or the Qubit assay, we arbitrarily decided to reverse transcribe four microliters of RNA for each sample as performed in our previous published work 41. After extraction Expression levels of miRNAs were analyzed using TaqMan MicroRNA assay probes. Real-time PCR for miR-204-5p, miR-199b-5p miR-579-3p, miR-9-5p miR-4443 and miR-4488 was assayed by the TaqMan Gene Expression.

### Statistical analysis

Data of circulating miRNAs were normalized using both the Global Mean normalization and the web-tool RefFinder (http://www.ciidirsinaloa.com.mx/RefFinder-master/) 52, 53. Descriptive statistics were calculated for all variables of interest. miRNA distributions among the different clinical responses were tested through the use of Student's T test model. For analysis purposes, ∆Ct miRNA values were dichotomized on the basis of the cut-off established using the receiver operating characteristics (ROC) curve considering Progressive Disease (PD) specific condition as the state variable. ROC curves were used in order to search for an optimal cut-off value with the highest sensitivity and specificity and Youden's index was performed in order to identify this value. Across various cut-off points, Youden's index maximized the difference between sensitivity and specificity and between real-positive and false-positive subjects. Thus, the optimal cut-off value was calculated. Overall Survival (OS) and Progression Free Survival (PFS) analyses were carried out by the Kaplan-Meier product-limit method. In particular Kaplan-Meier curves were obtained by a commercial scientific 2D graphing and statistics software called GraphPad Prism 8.0 54. The Log Rank test was used to prove whether any statistically significant differences between subgroups emerged. In addition, the same analyses were conducted in assessing the Ct raw values of the two miRNAs and their miRatio (*ratio miR-4488: miR-579-3p).* Finally, univariate and bivariate cox regression models were applied to obtain hazard risks (HR) with their relative 95% Confidence Intervals (95%CI) and all the results were plotted into forest plots 55 (Figure 4). These graphs were constructed to compare results of different univariate and bivariate analyses related to OS and PFS. The variables used in the models were the expression values of the two miRNAs (obtained through GM, RF methods or their raw Ct) adjusted for LDH values categorized according to the median value. P-values <0.05 were considered statistically significant. The SPSS (version 21.0) statistical program (SPSS Inc., Chicago, IL, USA) was used for our analyses.

## Discussion and conclusions

The identification of specific biomolecular signatures is a fundamental requirement for monitoring tumor progression and for predicting therapeutic responses. This is a rather important issue in BRAF mutated melanoma patients where two alternative options are available as first line therapy: targeted therapy with inhibitors of the MAPK pathway or immunotherapy with immune checkpoint inhibitors (ICI). Targeted therapy is frequently accompanied by the rapid development of drug resistance which results in a highly aggressive disease often associated with cross-resistance to ICI. Hence, an easily measurable biomarker capable of predicting failure to targeted therapy in-advance which may aid clinicians in making the best therapeutic decision.

MicroRNAs have been proposed as promising biomarkers in oncology for early diagnosis, prognosis and therapeutic response prediction including melanoma. In particular, specific miRNAs are able to differentiate melanoma patients from healthy subjects or distinguish between metastatic and non-metastatic melanoma patients. Numerous miRNAs were found dysregulated in tumor samples or in the bloodstream of patients with melanoma. Previous studies have identified a useful panel of cf-miRNAs (cf-miR-9-5p, cf-miR-145-5p, cf-miR-150-5p, cf-miR-155-5p and cf-miR-205-5p) to detect the presence of metastasis in patients with melanoma 56. Other studies have highlighted that miR-221 levels were increased in the serum of the metastatic patients and were correlated with tumor thickness reflecting a progression status of melanoma patients 15, 57. Another example worth mentioning concerns the miR-206, where high levels of this miRNA were detected in the serum of patients with advanced stages of melanoma associating miR-206 with aggressive disease progression and poor prognosis 15. Moreover, other miRNAs were found to be associated with resistance to therapies. In line with this, the same oncosuppressor miR-579-3p has been found to be strongly downregulated in tumor samples deriving from BRAF-mutated melanoma patients before and after the development of resistance to MAPKi 41. Furthermore, a study in which 25 BRAF-mutated melanoma patients treated with MAPKis were enrolled, found that the group with high levels of miR-497-5p measured by EVs isolated serum had a better PFS as compared to the group with low levels of the same miRNA. Again, in the same melanoma patients, increased levels of let-7g-5p during treatment with MAPKi obtained a major disease control (DC) as compared to group of patients with low levels 58. All these lines of evidence suggest that the expression level of specific miRNAs can be used as a powerful tool for both early detection/advanced stages of melanoma or follow-up of patients during treatment.

In line with this body of evidence, our research group carried out a retrospective analysis of 70 serum samples derived from BRAF-mutated melanoma patients treated with MAPKi therapy. In particular, we evaluated the expression levels of 6 cf-miRNAs as predictors of response to therapy before starting treatments. The goal of this study was to identify a possible "mini-signature" among these 6 miRNAs, able to discriminate *ab initio* of those patients who can benefit or not from MAPKi therapy. It is worthy to note that the 6 miRNAs are divided into three oncomiRs (miR-9-5p, miR-4443 and miR-4488) and three oncosuppressors (miR-579-3p, miR-204-5p and miR-199b-5p) found to be deregulated in plasma samples derived from 25 resistant BRAF-mutated melanoma patients in our previous published study 42. Interestingly, results obtained from our qRT-PCR analysis uncovered that significant deregulation of miR-579-3p (down-modulation) and miR-4488 (up-modulation) before starting therapy, is a peculiar feature for those patients who did not obtain an improvement in terms of PFS. A contrasting situation occurs in melanoma patients with low levels of miR-579-3p and high expression of miR-4488 as displayed in plotted Kaplan-Meier curves. Finally, Kaplan-Meier and log-rank testing were performed to also evaluate the effect of miR-579-3p/miR-4488 ratio (miRatio) on the prediction of MAPKi therapy. Pleasingly, the expression ratio of miR-4488/miR-579-3p demonstrated a better sensitivity and specificity for the treatment prediction with BRAFi and MEKi. In particular, high values of miRatio describe a worse prediction in terms of PFS. Quite the opposite was obtained with low values of the same ratio. Hence, we may assert that miR-4488/miR-579-3 ratio is able to predict an ab initio response to targeted therapy in BRAF-mutated melanoma patients. Finally, another interesting point to consider in the study is that also single Ct values of miR-579-3p and miR-4488 were able to significantly predict the development of drug resistance. This aspect should not be underestimated in terms of practicality. In fact, in evaluating the raw Ct values of the two miRNAs, all normalization analysis (based on ΔCt values) is bypassed. In clinical terms, treatment prediction for BRAF and MEK inhibitor therapies could be obtained both rapidly and easily. Based on these assumptions, the evaluation of miR-579-3p and miR-4488 expression in serum samples can work as useful biomarkers to predict whether targeted therapy may be or not be the appropriate treatment in BRAF-mutated melanoma patients.

Establishing the best first-line therapy can be crucial point in obtaining a greater patient survival. In conclusion, starting from these intriguing findings, two important steps need to be addressed in the near future which entail: 1) strengthening our discovery by enrolling a larger cohort of BRAF-mutated melanoma patients; 2) expanding the same type of study also to melanoma patients who are undergoing immunotherapy in order to identify miRNAs capable of predicting the response to immune checkpoint inhibitors.

## Supplementary Material

Supplementary figures and tables 1-3.

Supplementary table 4: DB pazienti target therapy Pascale patients 1.

Supplementary table 5: DB Target therapy (all T studi patients) Pascale patients 2.

Supplementary table 6: DB Target therapy TubeList Prog Melanomi IFO.

## Figures and Tables

**Table 1:** The table summarizes general characteristics of the 70 BRAF-mutated melanoma patients.

Characteristic	N (%)
**Number of cases**	70
**BRAF variant**	
V600E	57 (95%)
V600K	3 (5%)
V600 variant not available	10 (14%)
**Gender**	
Male	36 (51%)
Female	34 (49%)
**Age**	
Median (min-max)	54 yrs (26-89)
**Stage**	
III-IV	5 (7% III stage) 65 (93% IV stage)
**Breakdown of the patients**	CR = 12 (17%), PD = 22 (32%), PR = 24 (34%), SD = 12 (17%)
**Median LDH value**	0.95 30 (43% high LDH values) 30 (43% low LDH values) 10 (14% not available)

## Abbreviations

ICI: Immune Checkpoint Inhibitors
miRNAs: microRNAs
PFS: Progression Free Survival
OS: Overall Survival
LDH: Lactate dehydrogenase
MAPK: Mitogen-activated protein kinase
BRAF: v-raf murine sarcoma viral oncogene homolog B1
ROC: Receiver Operating Characteristics
GMN: Global Mean Normalization
RF: RefFinder
DC: Disease Control
PD: Progression Disease
CR: Complete Response
PR: Partial Response
AUC: Area Under Curve
