# Supplementary material for: Identification of a miRNA-based non-invasive predictive biomarker of response to target therapy in BRAF-mutant melanoma
Source: Theranostics. 2022 Oct 24;12(17):7420–30. doi: 10.7150/thno.77761 (PMC9691363; doi:10.7150/thno.77761)
Supplement: Supplementary file 1 — Supplementary figures and tables 1-3. [file thnov12p7420s1.pdf]

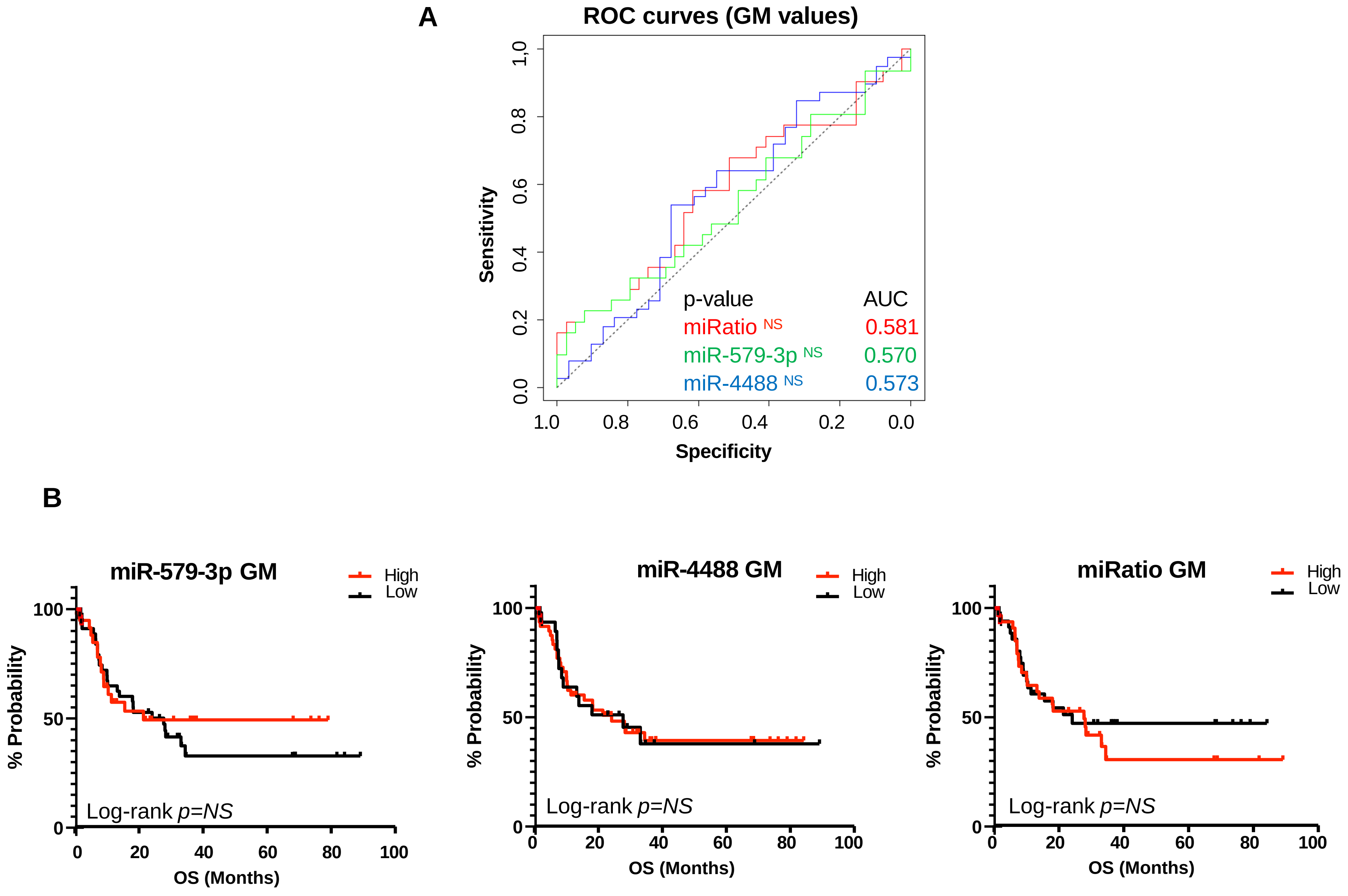

Figure S1. Results show that miR-579-3p, miR-4488 and miRatio were not able to predict overall survival (OS) in terms of ROC curves (A) and Kaplan-Meier graphs (B).

A

Method	1	2	3	4	5	6
Delta CT	miR-204-5p	miR-199b-5p	miR-4443	miR-579-3p	miR-9-5p	miR-4488
BestKeeper	miR-199b-5p	miR-4443	miR-204-5p	miR-579-3p	miR-9-5p	miR-4488
NormFinder	miR-199b-5p	miR-9-5p	miR-4443	miR-204-5p	miR-579-3p	miR-4488
Genorm	miR-199b-5p<br>mir-204-5p	-	miR-4443	miR-579-3p	miR-9-5p	miR-4488
**Recommended comprehensive raking**	**miR-199b-5p**	**miR-204-5p**	**miR-4443**	**miR-9-5p**	**miR-579-3p**	**miR-4488**

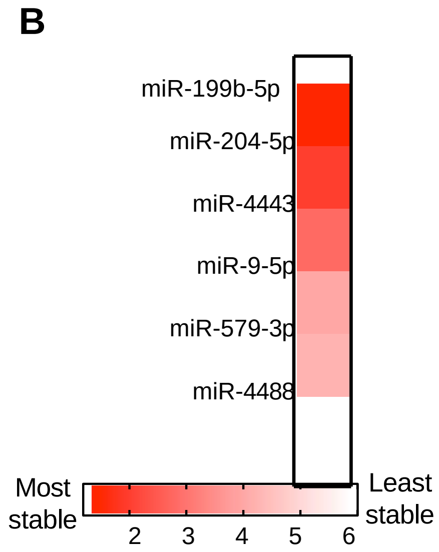

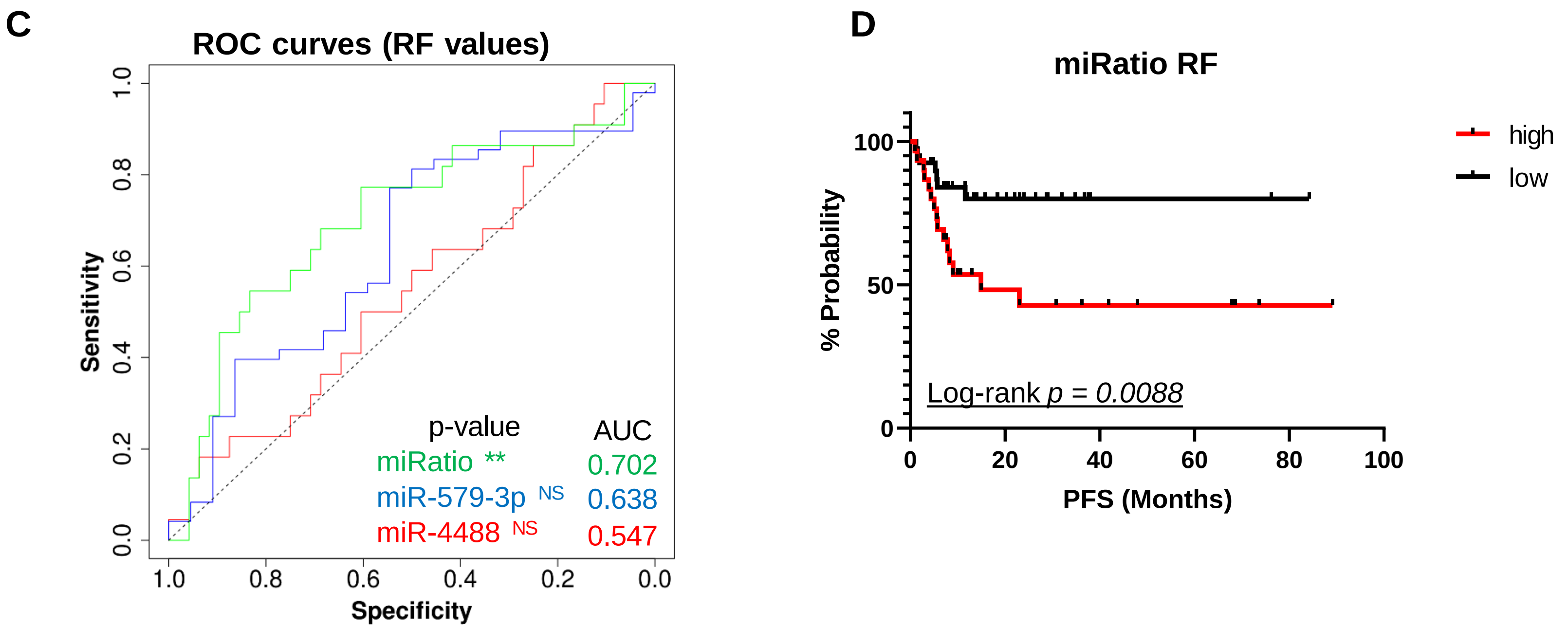

Figure S2. The table in figure A was obtained by RefFinder method. In detail this web-tool is able to establish which is the most stable miRNA among those take in consideration (A). In this specific case miR-199b-5p resulted to be the less fluctuant inside the 70 serum samples tested, therefore becoming the reference miRNA to perform our analyses as pictured in the color bas scale (B). The ROC curves obtained by RF method confirmed also in this case and in agreement with GM results i.e. the miRatio yielded the best AUC value (0,702) (C, red line). Finally also the Kaplan-meier graph significantly confirms what was obtained with GM analysis (D).

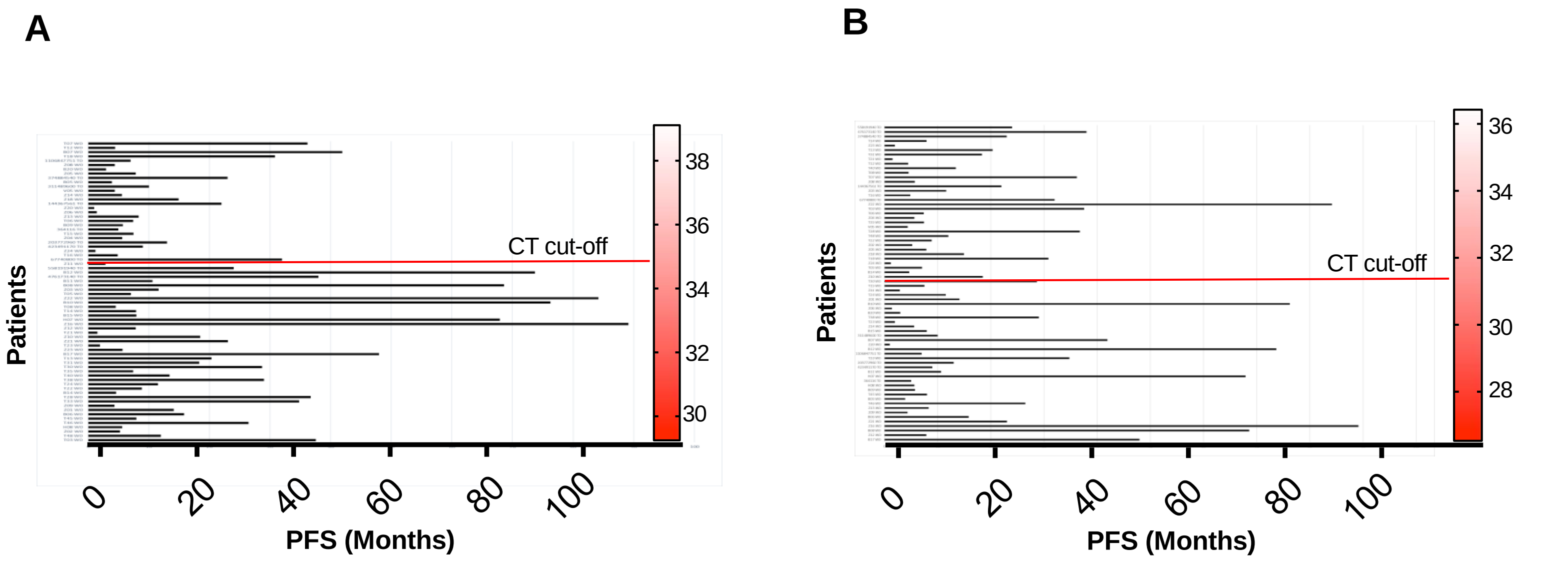

Figure S3. In the bar graphs melanoma patients were separated by CT cut-off values derived from ROC curves. The group of patients with CT cut-off values below median, i.e. the one composed of high miR-579-3p expression levels, is enriched for longer PFS as compared to the group of patients with CT cut-off values above median (A). An opposite trend were found regarding miR-4488 (B).

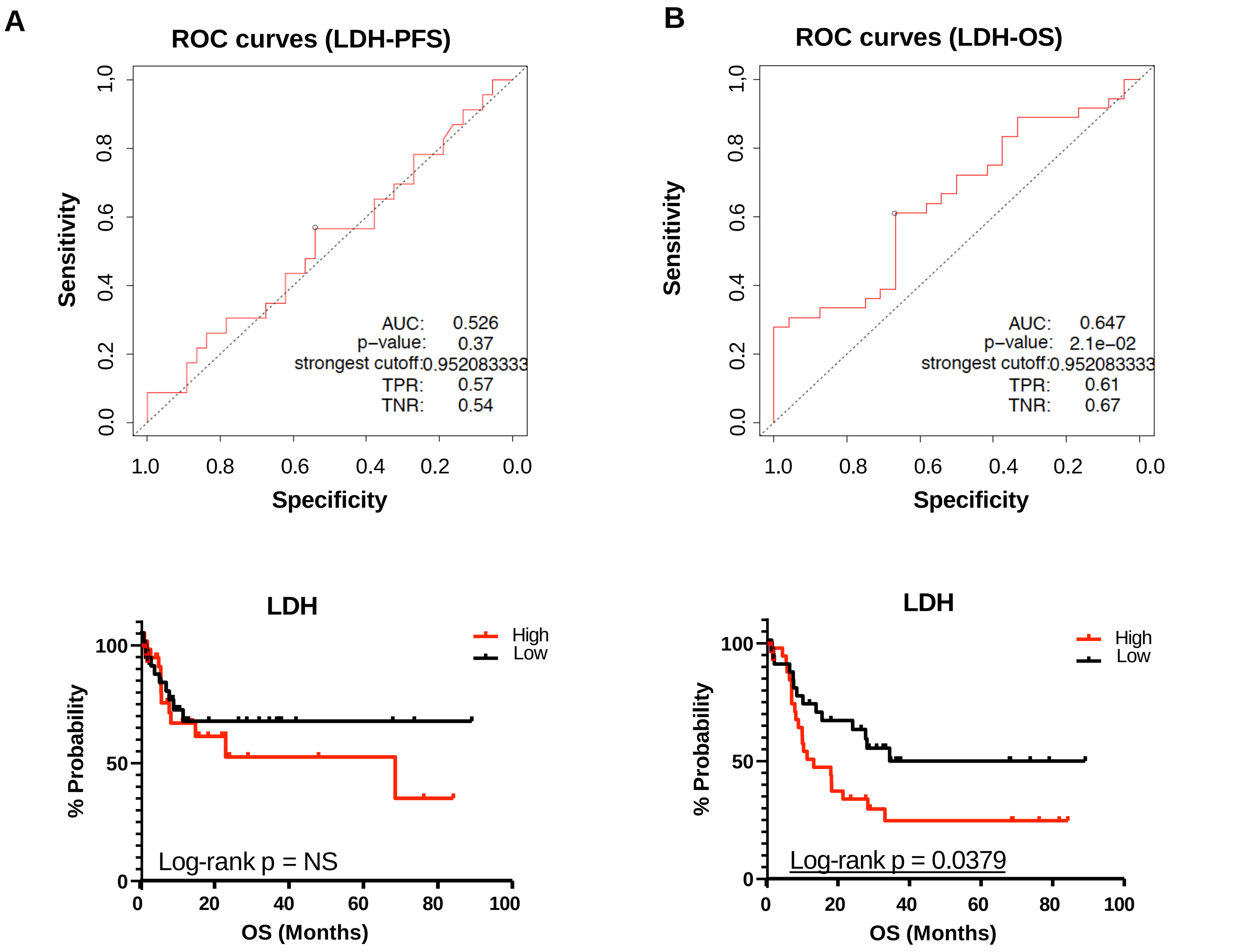

Figure S4. LDH was found not to be correlated with PFS. This aspect was confirmed by the ROC (A upper panel) and Kaplan-Meier curves (A bottom panel). On the reverse, as reported also in the literature, LDH were significantly found correlated with the OS as reported by ROC (B upper panel) and Kaplan-Meier curves (B bottom panel).

Global mean (GM)				
	**Area Under the Curve (AUC) (95%CI)**	**p-value**	**Test direction**	**Cut-off**
**miR-579-3p**	0.682 (0.65-0.73)	0.0046	Lower values indicate no response to treatment	0.34298
**miR-4488**	0.624 (0.55-0.65)	0.054	Higher values indicate no response to treatment	2.62067
**miRatio**	0.702 (0.7-0.7)	0.0026	Higher values indicate no response to treatment	7.76207

Table S1. In the table are reported AUC values, p-value and cut-off values of miR-579-3p, miR-4488 and miRatio respectively obtained from GM normalization data used for generating ROC curve.

Ref Finder (RF)				
	**Area Under the Curve (AUC) (95%CI)**	**p-value**	**Test direction**	**Cut-off**
**miR-579-3p**	0.638 (0.55-0.77)	0.03	Lower values indicate no response to treatment	0.45566
**miR-4488**	0.547 (0.50-0.60)	0.26	Higher values indicate no response to treatment	6.58984
**miRatio**	0.702 (0.7-0.7)	0.0026	Higher values indicate no response to treatment	7.762077

Table S2. In the table are reported AUC values, p-value and cut-off values of miR-579-3p, miR-4488 and miRatio respectively obtained from RF normalization data used for generating ROC curve.

Absolute CT				
	**Area Under the Curve (AUC) (95%CI)**	**p-value**	**Test direction**	**Cut-off**
**miR-579-3p**	0.679 (0.68-0.71)	0.0039	Higher values indicate no response to treatment	34.29130
**miR-4488**	0.610 (0.59-0.65)	0.062	Lower values indicate no response to treatment	30.84666
**miRatio**	0.697 (0.556-0.838)	0.0034	Higher values indicate no response to treatment	0.9278

Table S3. In the table are reported AUC values, p-value and cut-off values of miR-579-3p, miR-4488 and miRatio respectively obtained from Ct normalization data used for generating ROC curve.
